# Efficacy of Linezolid and Fosfomycin in Catheter-Related Biofilm Infection Caused by Methicillin-Resistant* Staphylococcus aureus*

**DOI:** 10.1155/2016/6413982

**Published:** 2016-06-05

**Authors:** Dong Chai, Xu Liu, Rui Wang, Yan Bai, Yun Cai

**Affiliations:** ^1^Department of Pharmaceutical Care, PLA General Hospital, 28 Fu Xing Road, Beijing 100853, China; ^2^Department of Clinical Pharmacology, PLA General Hospital, 28 Fu Xing Road, Beijing 100853, China; ^3^Translational Medical Center, PLA General Hospital, 28 Fu Xing Road, Beijing 100853, China

## Abstract

As long-standing clinical problems, catheter-related infections and other chronic biofilm infections are more difficult to treat due to the high antibiotic resistance of biofilm. Therefore, new treatments are needed for more effective bacteria clearance. In this study, we evaluated the antibacterial activities of several common antibiotics alone and their combinations against biofilm-embedded methicillin-resistant* staphylococcus aureus* (MRSA) infections, both* in vitro* and* in vivo*. In brief, fosfomycin, levofloxacin, and rifampin alone or in combination with linezolid were tested* in vitro* against planktonic and biofilm-embedded MRSA infection in three MRSA stains. The synergistic effects between linezolid and the other three antibiotics were assessed by fractional inhibitory concentration index (FICI) and time-kill curves, where the combination of linezolid plus fosfomycin showed the best synergistic effect in all strains. For further evaluation* in vivo*, we applied the combination of linezolid and fosfomycin in a catheter-related biofilm rat model and found that viable bacteria counts in biofilm were significantly reduced after treatment (*P* < 0.05). In summary, we have shown here that the combination of linezolid and fosfomycin treatment had improved therapeutic effects on biofilm-embedded MRSA infection both* in vitro* and* in vivo*, which provided important basis for new clinical therapy development.

## 1. Introduction

Methicillin-resistant* Staphylococcus aureus* (MRSA) is a common nosocomial pathogen that causes many catheter-related infections and other chronic biofilm infections [1]. Vancomycin has been considered as the standard treatment for invasive MRSA infection, but the recent evidence is suggesting its poor efficacy, mainly caused by the formation of biofilm [2]. With high tolerance to antibiotics, biofilms can serve as protective niches for MRSA within host, which further increases the minimum inhibitory concentration (MIC) of MRSA. Moreover, the small colony variants within biofilms may also play a role in antibiotic resistance [3].

To solve this problem, a potential way is to reassess the older generations of antibiotics and investigate the novel combinations of existing agents [4]. For example, Corvec et al. [5] found that the combinations of colistin with tigecycline or fosfomycin had significantly improved antibacterial activities against biofilm infection caused by fluoroquinolone-resistant* Escherichia coli*. In another study, Oliva et al. [6] showed the synergistic effect of fosfomycin plus gentamicin in antibacterial activity against* Enterococcus faecalis* biofilm.

Linezolid is the first synthetic oxazolidinone antibiotic that blocks protein synthesis by preventing the formation of the initiation complex. It has been approved as an alternative drug for the treatment of staphylococcal skin and soft-tissue infections or pneumonia [7]. Chronic osteomyelitis is a multifaceted bacterial infection requiring surgery in concomitance to antibiotics for treatment. Linezolid has a lot of advantages in treatment of chronic osteomyelitis, such as high bone-serum concentration ratio, having high bioavailability, and being minimally affected by renal dysfunction [8]. Linezolid monotherapy has been reported to have inhibitory effects against biofilm-embedded MRSA [2, 9]. However, the doses used in these studies were relatively high, which might lead to linezolid-associated adverse drug reactions, such as dose-dependent thrombocytopenia [10]. Fosfomycin is a broad-spectrum bactericidal drug against both Gram-positive and Gram-negative bacteria. It has good activity against both extended-spectrum beta-lactamases and carbapenemase-producing* Enterobacteriaceae* and >90% multidrug resistant* P*.* aeruginosa* [11]. For MRSA, fosfomycin also has high activity as 87.9% (4240/4892 isolates) cumulative susceptibility rate [12]. Linezolid combined with fosfomycin, rifampin, and levofloxacin has been used to effectively treat planktonic or biofilm MRSA infections* in vitro* [2, 9, 13]. Therefore, the* in vivo* studies need to be conducted to evaluate the potential of linezolid combination regimens in clinical treatments against biofilm-associated MRSA infections. In our study, the effects of fosfomycin, rifampin, and levofloxacin alone or in combination with linezolid on viable bacterial counts in biofilms were evaluated both* in vitro* and* in vivo*, which provided thorough information about the antibiofilm effects of linezolid combination regimens.

## 2. Methods

### 2.1. Strains and Agents

In the previous study, we found 98.04% synergy of the combination of linezolid and fosfomycin against 102 planktonic MRSA strains by fractional inhibitory concentration index (FICI)* in vitro* [7]. Therefore, 3 MRSA strains with numbers 154311, 152898, and 159228 were randomly selected from the 102 strains for both* in vitro* and* in vivo* studies. The strains were clinically isolated from PLA General Hospital and were identified by the automated VITEK-2 system (bioMérieux, Marcy l'Etoile, France) with a rapid latex agglutination test.* Staphylococcus aureus* ATCC 25923 was used as the quality control strain.

Fosfomycin, rifampin, and levofloxacin (National Institute for Food and Drug Control, Beijing, China) were used for the* in vitro* study. Fosfomycin sodium (Northeast Pharm, Shenyang, China) and linezolid (for injection, Pfizer, Madison, USA) were used in the biofilm-infection rat model.

### 2.2. Fractional Inhibitory Concentration Assay

Synergy of antibiotics was assessed using the checkerboard broth microdilution method assays as described previously [7]. In brief, 96-well plates were set up with increasing concentrations of linezolid in the horizontal wells and fosfomycin, rifampin, and levofloxacin in the vertical wells. Each well was inoculated with 5 × 10^5^ cfu/mL MRSA prepared in broth. Three MRSA strains (154311, 152898, and 159228) were determined in FIC assay. The plates were incubated at 37°C for 24 hours and visually inspected for turbidity to determine the growth. Experiments were performed in triplicate. The interactions between two tested antimicrobials were evaluated by FICI, calculated as follows: FICI = (MIC of drug A in combination/MIC of drug A alone) + (MIC of drug B in combination/MIC of drug B alone). The FICI was interpreted as follows: FICI ≤ 0.5, synergy; 0.5 < FICI ≤ 4.0, indifference; FICI > 4.0, antagonism [7].

### 2.3. Time-Kill Assay

Time-kill experiments were performed on MRSA 154311 according to the Clinical and Laboratory Standards Institute (CLSI) methodology [14]. Briefly, bacterial suspensions were diluted to 1.5 × 10^5^ cfu/mL in 10 mL of Muller-Hinton Broth (MHB) (BD Difco, Franklin Lakes, USA) for inoculation. The concentrations of fosfomycin were adjusted to 1/16x (4 mg/L), 1/8x (8 mg/L), 1/4x MIC (16 mg/L), and 1/2x MIC (32 mg/L). Each concentration was tested alone or in combination with 1/2x MIC (1 mg/L) linezolid for time-kill curve assays. Bacterial counts were done at 0, 2, 4, 8, 12, and 24 h by spreading 10-fold serial dilutions onto Muller-Hinton agar plates (BD Difco, Franklin Lakes, USA). The experiments described above were repeated three times. Synergy was defined as more than 2 lg 10 cfu/mL decrease between the combination and its most active constituent after 24 h (at least one of the drugs must be present at a concentration that does not affect the growth curve of the tested organism), and the number of viable organisms in the presence of the combination must be ≥2 lg 10 cfu/mL below the starting inoculum [15].

### 2.4. Synergy Test in Biofilm* In Vitro*

Synergy test was performed on all MRSA strains. Biofilm was cultivated on disks in 24-well plates. Bacterial suspension was adjusted to 0.5 McFarland (1.5 × 10^8^ cfu/mL). 2 mL of MHB II and 100 *μ*L of bacterial suspension were added to each well containing 3 catheter disks (diameter, 0.5 cm). The plates were then incubated at 37°C for 5 days, and MHB II was renewed daily. The catheter disk with biofilm model used in this study was based on several published articles that focused on antibiofilm effects of some antimicrobial agents [16, 17]. The produced catheter disks were then cut and put into Portex endotracheal tubes (Smiths Medical Ltd, Hythe, UK) using a prototype mold with diameter of 0.5 cm. The endotracheal tube was made of transparent polyvinyl chloride (PVC). The tests were performed in 96-well plates, and each well contained 200 *μ*L linezolid at 1/2x MIC, 1/4x MIC, and 1/8x MIC, together with fosfomycin or levofloxacin at 1/2x MIC, 1/4x MIC, and 1/8x MIC, and rifampin at a fixed concentration of 1 mg/L, according to previous studies [9]. Biofilm disks were randomly selected from the 24-well plates, rinsed with physiological saline to remove planktonic bacteria, and then placed into 96-well plates. Each well contained one disk. After 24 h of treatment at 37°C, these disks were taken out and washed with saline three times to remove planktonic bacteria. The adherent bacteria were collected from disks by using an ultrasonic cleaning bath in 10 min. The bacterial solution was vigorously mixed and plated on agar plates as 10-fold serial dilutions and cultured for 16~24 h. The colony numbers between 30 and 300 per plate were considered as good results, and this standard was also used for the* in vivo* study in biofilm-infection rat model. Each treatment had six catheters. Bacteria counts were repeated three times.

### 2.5. Synergy Test in Biofilm-Infection Rat Model

MRSA 154311 biofilm was grown on catheters (length = 3.5 cm, *r* = 0.4 cm). Each catheter was placed into a 5 mL tube containing 3 mL MHB for culture. 100 *μ*L MRSA suspension at 0.5 McFarland which includes 1.5 × 10^7^ bacteria cells was inoculated into each tube (5 × 10^5^ cfu/mL) and cultured for 5 days at 37°C. The catheters were the same as those used in* in vitro* experiments. Twenty-eight Wistar male rats, weighing 200–250 g, were anesthetized with intraperitoneal injection of 4% chloral hydrate at 0.01 mL/g. Then the catheters with biofilms were rinsed with sterile physiological saline solution and implanted at dorsal midline, as previously described [18]. Rats were randomly divided into four groups with 7 rats in each group: group LIN (linezolid 40 mg/kg/12 h); group FOS (fosfomycin, 300 mg/kg/12 h); group LIN + FOS (linezolid 40 mg/kg/12 h plus fosfomycin 300 mg/kg/12 h); and group control (saline/12 h). The dosage and delivery methods were according to previous studies [19, 20]. After implantation, the antimicrobial agents were immediately injected intraperitoneally. At the end of antimicrobials therapy (7 days after), the implanted catheters in all four groups were taken out to examine the bacterial counts in biofilms. The method for counting bacteria in biofilm was the same as that in the* in vitro* synergy test. Real-time monitoring of WBCs changes in rats reflected infection conditions. Twenty microliters of blood was drawn from the tail vein of each rat before infection and at days 1, 3, and 5 during therapy. WBCs were examined using an animal blood analytical instrument (Mindray BC-2800Vet, Shenzhen, China). The data are presented as mean ± standard deviation (SD) (*n* = 7).

### 2.6. Statistical Analysis

All the data were presented as mean ± standard deviation. Differences in lg cfu counts among groups were assessed by one-way analysis of variance (ANOVA). SPSS 12.0 was used for statistical analyses. Differences with *P* < 0.05 were considered significant.

## 3. Result

### 3.1. Synergistic Effect of Linezolid and Fosfomycin on Planktonic MRSA* In Vitro*

**Figure 1:**
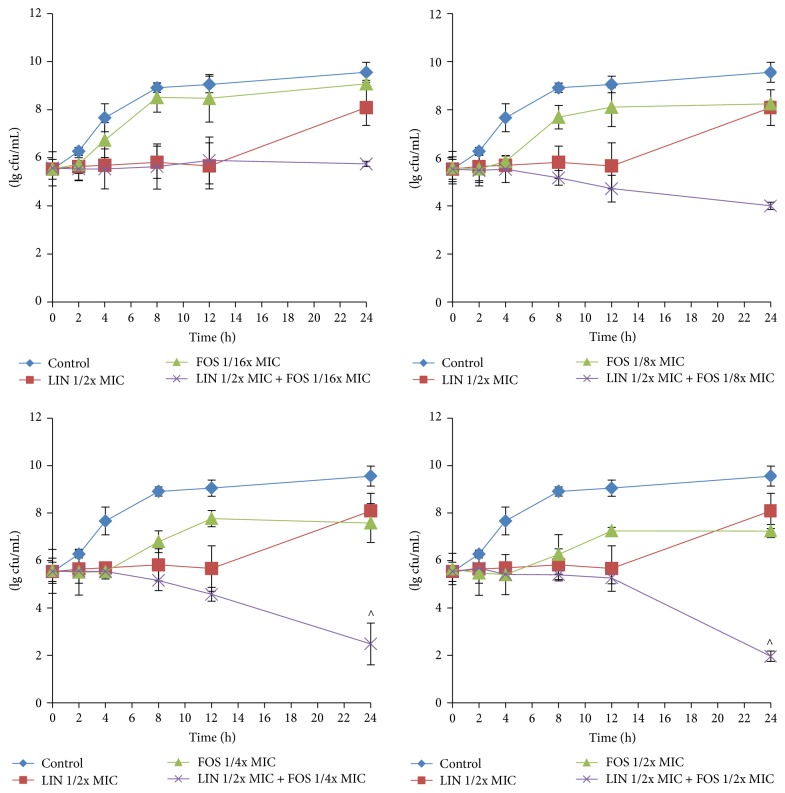
Time-kill curves of linezolid plus fosfomycin* in vitro*. 154311 MRSA strain was used in this assay. The concentrations of antimicrobial agents in each group were LIN, 1/2x MIC (1 mg/L) linezolid; FOS, 1/16x MIC (4 mg/L), 1/8x MIC (8 mg/L), 1/4x MIC (16 mg/L), and 1/2x MIC (32 mg/L) fosfomycin; LIN + FOS, 1/2x MIC (1 mg/L) linezolid plus 1/16x (4 mg/L), 1/8x (8 mg/L), 1/4x (16 mg/L), and 1/2x MIC (32 mg/L) fosfomycin; control: broth alone. Data are presented as mean ± SD. All experiments were repeated three times.

The MICs and FICI of the tested antimicrobial agents were summarized in Table 1. For number 154311 strain, both linezolid plus fosfomycin and linezolid plus levofloxacin showed synergistic effect revealed by FICI. The MICs of linezolid and fosfomycin were 2 mg/L and 64 mg/L, respectively, and the FICI 0.375 was obtained with 0.5 mg/L linezolid (1/4x MIC) and 8 mg/L fosfomycin (1/8x MIC). To confirm the synergistic activity between linezolid and fosfomycin, time-kill experiments were performed. As shown in Figure 1, all the combination groups had significantly reduced bacterial counts at 24 h time point compared to either linezolid or fosfomycin alone (*P* < 0.05). In addition, 1/2x MIC (1 mg/L) of linezolid combined with 1/4x MIC (16 mg/L) or 1/2x MIC (32 mg/L) of fosfomycin exhibited synergistic effect. These results indicated that the linezolid-fosfomycin combination had improved efficiency against planktonic MRSA.

### 3.2. Synergistic Effect of Linezolid and Fosfomycin on MRSA Biofilm* In Vitro*

Table 2 summarized the test results of linezolid in combination with fosfomycin, levofloxacin, and rifampin against three kinds of MRSA biofilms. Linezolid plus levofloxacin or rifampin showed reduced viable bacterial counts in biofilm for two stains. And linezolid at 1/2x MIC plus fosfomycin at 1/2x MIC exhibited significantly reduced viable bacterial counts in biofilm for all stains, with the biggest effect on strain 154311 cultured biofilm. These results also suggested that the synergistic effect of linezolid plus fosfomycin was dose-dependent. Based on these* in vitro* results, we chose the combination of linezolid plus fosfomycin and further investigated its antibacterial effect against strain 154311 MRSA biofilm* in vivo*.

### 3.3. Synergistic Effect of Linezolid and Fosfomycin on MRSA Biofilm* In Vivo*

At 7 days after infection, the colony counts in biofilms of control, LIN, FOS, and LIN + FOS groups were 7.46 ± 0.35, 5.97 ± 0.97, 5.26 ± 0.29, and 2.98 ± 0.40 lg cfu/mL, respectively. Both linezolid and fosfomycin alone showed decreased colony counts compared to control group (*P* < 0.05), and LIN + FOS group, with the colony counts reduced by 4.5 lg cfu/mL, showed significant lower colony numbers than any other groups (*P* < 0.05).

To examine the effects of the drugs on inflammation of the biofilm-infected rats, the values and normal rate of WBCs (defined as the number of rats whose WBCs counts ranged from 2.9 to 15.3 × 10^9^/L divided by the total number of rats in group) were evaluated before infection and at 1, 3, and 5 days after infection. WBC results were as follows: control group: 8.15 ± 2.21 (100%), 6.88 ± 2.08 (100%), 10 ± 2.1 (100%), and 16.42 ± 4.89 (33.33%); LIN: 10.09 ± 3.04 (100%), 9.44 ± 4.64 (100%), 11.2 ± 2.58 (100%), and 14.54 ± 2.61 (71.43%); FOS: 8.30 ± 1.74 (100%), 8.40 ± 2.35 (100%), 9.81 ± 1.27 (100%), and 16.2 ± 2.11 (57.14%); LIN + FOS: 9.01 ± 3.11 (100%), 9.96 ± 2.57 (100%), 9.96 ± 2.05 (100%), and 13.47 ± 1.96 (85.71%). WBCs of the four groups were all continuously increasing from day 0 to day 5. At day 5, the WBCs of LIN + FOS group was 13.47 ± 1.96, lower than the other groups. But no significant difference among groups was observed.

## 4. Discussion

In this study, the combination of linezolid and fosfomycin displayed the most effective antibacterial effect against MRSA* in vitro*, compared with other combinations. A synergistic effect of linezolid and fosfomycin has been observed* in vitro* in previous studies. Pachón-Ibáñez et al. evaluated the efficacy of the combination of fosfomycin and linezolid against* Staphylococcus aureus* strain using time-kill curve. Compared with the initial bacterial counts, a nearly 3 lg cfu/mL reduction at 24 h time point was observed from the treatment of 1x MIC linezolid plus 1x MIC fosfomycin [21]. Similar results were also seen in our study. More than 3 lg cfu/mL decrease compared to starting inoculum at 24 h point was observed from the treatment of 1/2x MIC (1 mg/L) linezolid plus 1/4x MIC (16 mg/L) or 1/2x MIC (32 mg/L) fosfomycin.

Our study also demonstrates that linezolid plus fosfomycin is an effective combination against MRSA biofilm* in vitro*. Several mechanisms have been proposed to explain why only a few antibiotics are capable of fighting against biofilms. Fosfomycin has been reported to be involved in some combination therapies that showed increased therapeutic effects. The low molecular weight of fosfomycin could partially explain its facilitated effect in antibacterial activity against biofilm-related organisms [2]. Fosfomycin was reported to destroy or change the outer layer of bacteria and thus inhibit the first step of cell wall synthesis. As a result, linezolid can easily get into the cells and act synergistically with fosfomycin [19]. In addition, fosfomycin alone treatment against strain 154311 biofilms showed a concentration-dependent activity from 1/8x MIC (8 mg/L) to 1/2x MIC (32 mg/L), which may lead to the concentration-dependent manner of linezolid plus fosfomycin treatment in this study.

For* in vivo* studies, Mihailescu et al. and Baldoni et al. [22, 23] used a guinea pig model to evaluate the effects of fosfomycin and linezolid, alone or in combination with rifampin, against MRSA biofilm infection. Animals with cage implant infections were treated with antimicrobial agents. The efficacy was evaluated by cure rate defined as the number of cage cultures without MRSA growth divided by the total number of cages in the treatment group. Antimicrobial agent alone was unable to eradicate biofilm MRSA from cages. But linezolid plus rifampin achieved 50% to 60% cure rate, and fosfomycin plus rifampin achieved 83% cure rate. We also found consistent results in our study. Only a decrease of about 2 lg cfu/mL in bacterial counts was observed in fosfomycin or linezolid alone groups compared to control group, but a much larger decrease of 4.5 lg cfu/mL was shown in linezolid plus fosfomycin group. However, no catheter cultures without MRSA growth were observed in linezolid plus fosfomycin treatment in our study. This might be due to the difference in biofilm incubation time. In their studies, they percutaneously inoculated MRSA planktonic bacteria into cages after implanting sterile cages in guinea pigs, and antimicrobial treatment was initiated 24 h or 3 days after infection. However, we investigated the antibacterial efficacy on mature biofilms, which were incubated for 5 days before treatment. Mature biofilm was more resistant than those in the initial stage, which may cause the complete clearance of infection in other studies but not ours [24].

White blood cells are a part of the immune system that helps body fight infection. The endotoxins produced by bacteria may elicit immune responses [25], which result in WBC production. In this study, we found that the combined agents had higher normal rates (85.71%) than other groups, although they were not significantly different. This could be due to the better antibacterial effects of combination agents that led to decreased bacterial counts and reduced endotoxins production, which in turn resulted in less WBC and weaker host responses.

As reported previously, high concentrations of antibiotics might be necessary to treat biofilm-related infections. However, high doses of linezolid or fosfomycin can cause some adverse effects, such as thrombocytopenia and peripheral neuropathy [10]. The treatment of linezolid in combination with fosfomycin could decrease the concentration of both. Therefore, it can not only enhance the treatment efficacy but also reduce the risk of adverse effects.

In conclusion, we found linezolid plus fosfomycin combination showed antibiofilm effect against MRSA strains both* in vitro* and* in vivo*. These results provided important basis for developing new regimens to treat patients with biofilm-associated MRSA infections, especially for catheter-related infections.

## Figures and Tables

**Table 1:** Minimum inhibitory concentration (MIC) and fractional inhibitory concentration index (FICI) of antibiotics against three methicillin-resistant *staphylococcus aureus* strains.

Strain	MIC				FICI		
	LIN	FOS	LEV	RIF	LIN-FOS	LIN-LEV	LIN-RIF
154311	2 (S)	64 (R)	0.25 (S)	>128 (R)	0.375 (synergism)	0.25 (synergism)	>1 (no interaction or antagonism)
152898	2 (S)	32 (R)	0.25 (S)	>128 (R)	0.5 (synergism)	0.75 (no interaction)	>1 (no interaction or antagonism)
159228	2 (S)	64 (R)	0.25 (S)	>128 (R)	0.375 (synergism)	0.75 (no interaction)	>1 (no interaction or antagonism)

LIN: linezolid; FOS: fosfomycin; LEV: levofloxacin; RIF: rifampin

S: susceptible; R: resistant. MIC was according to CLSI standards: Clinical and Laboratory Standards Institute.

FICI = FIC_A_ + FIC_B_ = (MIC A_combiantion_/MIC A_alone_) + (MIC B_combiantion_/MIC B_alone_) = (MIC of drug A in combination/MIC of drug A alone) + (MIC of drug B in combination/MIC of drug B alone).

FICI values were interpreted as follows: synergy, FICI ≤ 0.5; no interaction, 0.5 < FICI ≤ 4.0; antagonism, FICI > 4.0.

**Table 2:** Bacterial counts changes of the three biofilm kinds cultured by methicillin-resistant *Staphylococcus aureus *after 24 hours of exposure to three antibiotics alone or in combination with linezolid, compared to the viable bacterial counts in biofilm without antibiotic exposure.

Drugs	Bacterial counts (cfu/mL)					
	154311		152898		159228	
	Mean	SD	Mean	SD	Mean	SD
No antibiotics	6.50	0.17	6.89	0.02	7.16	0.13
LIN 1/8x MIC	6.48	0.12	6.88	0.03	6.71	0.22
FOS 1/8x MIC	6.07	0.12	6.26	0.13	7.23	0.23
LIN 1/8x MIC + FOS 1/8x MIC	5.64^*∗*^	0.09	6.14	0.33	6.75	0.50
LIN 1/4x MIC	6.24	0.13	6.74	0.02	6.44^*∗*^	0.10
FOS 1/4x MIC	5.14^*∗*^	0.09	6.15	0.15	7.13	0.12
LIN 1/4x MIC + FOS 1/4x MIC	4.09^*∗*^	0.22	5.97^*∗*^	0.10	6.96	0.06
LIN 1/2x MIC	5.74^*∗*^	0.14	6.39	0.05	6.20^*∗*^	0.09
FOS 1/2x MIC	4.87^*∗*^	0.08	6.28	0.06	6.75	0.30
LIN 1/2x MIC + FOS 1/2x MIC	1.86^*∗*^	0.10	5.30^*∗*^	0.09	6.40^*∗*^	0.07
LEV 1/8x MIC	6.49	0.17	6.58	0.10	6.79	0.27
LIN 1/8x MIC + LEV 1/8x MIC	6.20	0.24	7.12	0.17	7.20	0.26
LEV 1/4x MIC	6.14	0.06	6.53	0.15	7.19	0.23
LIN 1/4x MIC + LEV 1/4x MIC	5.34^*∗*^	0.08	6.50	0.11	6.87	0.27
LEV 1/2x MIC	6.38	0.17	6.43	0.26	7.23	0.23
LIN 1/2x MIC + LEV 1/2x MIC	5.35^*∗*^	0.12	6.56	0.12	6.42^*∗*^	0.09
RIF (1 mg/L)	6.04	0.37	6.70	0.07	6.67	0.34
LIN 1/8x MIC + RIF (1 mg/L)	6.30	0.14	6.78	0.09	6.84	0.25
LIN 1/4x MIC + RIF (1 mg/L)	5.36^*∗*^	0.28	6.71	0.05	6.86	0.11
LIN 1/2x MIC + RIF (1 mg/L)	5.50^*∗*^	0.16	6.48	0.24	6.47^*∗*^	0.32

LIN: linezolid; FOS: fosfomycin; LEV: levofloxacin; RIF: rifampin

Data are shown as means ± standard deviations. An asterisk (*∗*) indicates significant difference *P* < 0.05.
